# Predictive and prognostic role of mean platelet volume in patients with first-ever acute ischemic stroke

**DOI:** 10.17712/nsj.2017.2.20160330

**Published:** 2017-04

**Authors:** Ugur Lok, Umut Gulacti, Burcu Ekmekci, Taner Bulut, Murat Celik

**Affiliations:** *From the Departments of Emergency Medicine (Lok, Gulacti, Celik), Neurology (Ekmekci), and Radiology (Bulut), Faculty of Medicine, Adiyaman University, Adiyaman, Turkey*

## Abstract

**Objective:**

To investigate any possible effects of mean platelet volume (MPV) on short-term stroke prognosis and functional outcome in patients with first-ever acute ischemic stroke (FEAIS).

**Methods:**

This retrospective cross-sectional study included 798 FEAIS patients admitted to the emergency department of a tertiary care hospital in Adiyaman, Turkey between January 2013 and June 2015. The data were evaluated according to whether alive or dead, MPV levels, modified Rankin scale (MRS) scores, National Institutes of Health Stroke Scale (NIHSS) scores. The patients were divided into 3 groups based on MPV level as 4.4-7.4 fL, 7.5-10.4 fL, higher than 10.4 fL.

**Results:**

A total of 250 patients with FEAIS were included in the study. In both those who survived and those who died, the area under the curve related to hospitalization days, time interval of venipuncture (TIV), and MPV measurements was not statistically significant (*p*>0.05). The 3 MPV groups showed no significant differences in terms of MRS score, median NIHSS score, hospitalization, and TIV. In subgroups based on MRS scores, there were no statistically significant differences according to median latency (*p*=0.087), median hospitalization (*p*=0.394), TIV (*p*=0.201), and MPV levels (*p*=0.847). Furthermore, there were no differences in MPV levels between the MRS based groups (*p*=0.527).

**Conclusion:**

The results showed that MPV was not a significantly associated and reliable marker for the prediction of prognosis or functional outcome of FEAIS attack.

Acute ischemic stroke (AIS) has been clinically defined as a sudden-onset loss of focal cerebral function that persists for more than 24 hours. Worldwide, it is the second most prevalent reason for death and the most common reason for long-term incapability. The mechanism of stroke is largely described as any disease course that impedes oxygen and nutrient rich blood stream to the brain tissues and leads to focal neurologic syndromes that are related to interruption of substrates, such as oxygen and glucose, essential for production of high-energy phosphate compounds and the existence of mediators of secondary ischemic cellular injury. When a cell consumes the oxygen and substrates, death of cell are activated via pro-apoptotic genes, cell loses vitality and dies eventually.^1^-^3^ The most common disease process underlying AIS is atherothrombotic pathogenesis. The equilibrium between fibrin construction, platelet activation, and fibrinolysis, plays a pivotal role in atherothrombotic events in AIS, and may be important in the prognosis and progression of stroke. Platelets in particular play an important part in the formation of cerebral atherothrombotic events and ischemic processes, which encompass adhesion, release reaction, and aggregation of platelets.^4^,^5^ Circulating platelets are heterogeneous with regard to their size, density, and reactivity, and platelet volume indices are biomarkers of degree of platelet activation that are thought to be associated with systemic inflammatory responses. These indices include platelet count (PC), mean platelet volume (MPV), and platelet distribution width (PDW). It is generally known that large platelets contain a greater number of dense granules and a higher level of platelet aggregation markers, such as b-thromboglobulin, thromboxane B2, the prothrombotic substance, serotonin, and procoagulatory surface proteins, such as glycoprotein IIIA and P-selectin, which are not normally found in plasma, and are metabolically and enzymatically more active than those that are small. Increased MPV decreases the inhibitory effectiveness of prostaglandin on both platelet aggregation and the release of reactin. It is accepted that increased MPV may be an indicator of increased platelet activation, and may thus be related to the severity and prognosis of stroke; the larger the MPV, the worse the outcome. It has been postulated that the MPV that is determined early post-stroke (for example, within the first 48 hours) largely represents the pre-stroke status.^4^-^10^ International literature has controversial reports regarding the effect of MPV on stroke events. Although it has generally been asserted that platelet volumes are elevated in stroke, no previous study has examined the association between MPV and first-ever acute ischemic stroke (FEAIS) with regard to prognosis and stroke severity. We hypothesize that increased MPV may be a prehospital indicator of poor outcome for acute stroke. Thus, in the present study we evaluated the prognostic and predictive significance of MPV in people experiencing FEAIS, using a short-term follow up period in the Emergency Department (ED).

## Methods

### Study design

This retrospective cross-sectional study was conducted between January 2013 and June 2015 using the emergency department (ED) and stroke center (SC) database of the tertiary care Adiyaman University Education and Training Hospital, Adiyaman, Turkey. The study was approved by the local ethics committee and was conducted according to the principles of the Helsinki Declaration.

### Study protocol

The study data were retrospectively provided from an official hospital electronic patient database system that used the International Classification of Diseases-10 (ICD-10) coding system in accordance with stroke. The demographic features of the patients, MPV test results, co-morbidities, premorbid states, subtype of acute stroke, latency, National Institutes of Health Stroke Scale (NIHSS) and modified Rankin scale (MRS) scores, initial symptoms, hospitalization period (days), time interval of venipuncture (TIV), time between admission to emergency service and blood puncturing (hour), etiology, and the Trial of Org 10172 in Acute Stroke Treatment (TOAST) classification, complications that occurred during the admission period, and the definitive diagnosis, and outcome of the patients were recorded on the form. Outcome measures were assessed by the NIHSS score on admission, MRS scores at discharge, and hospitalization days. The ICD-10 codes in the diagnosis of acute stroke, which are the standard diagnostic tools for epidemiology, health management, and clinical purposes, were defined according to the 1990 focused update of the World Health Organization (WHO), which has been in use in WHO Member States since 1994. Final diagnosis of FEIAS was performed by a senior neurologist and radiologist.

### Study setting and population

Patients who were admitted to the ED with AIS during the study period were retrospectively documented. Patient eligibility for the study was identified by the attending emergency physician and a senior neurologist.

### Inclusion and exclusion criteria

Adult patients with FEAIS who were admitted to the ED were included in study. Patients for whom there were missing data or an inappropriate identification code, or those who were inappropriate for the study in any way were excluded. Additionally, patients presenting to the ED with all types of hemorrhagic stroke, such as subarachnoid, subdural, epidural, intraparenchymal, and intraventricular hemorrhages, a history of prior stroke attack at any time, transient ischemic attack (TIA), any malignancy, chronic inflammatory disease (for example, connective tissue disorders, such as vasculitis, rheumatoid arthritis, systemic lupus erythematosus, renal and hepatic insufficiency, and pancreatitis), organ transplantation, or other immunosuppressive etiologies, a history of previous thrombosis, hemoglobinopathies, and patients with fever at presentation as MPV may have been affected were also excluded.

The FEAIS patients primarily were assessed according to mortality (death or alive), MPV levels, and MRS score. The first group was formed of patients with an MPV of 4.4-7.4 fL; the second group was formed of patients with an MPV of 7.5-10.4 fL; and the third group was formed of patients with an MPV that was higher than 10.4 fL. The MRS scores were defined as MRS-1 = no significant disability, despite symptoms, that is, they were capable of carrying out all their usual duties and activities; MRS-2 = slight disability, that is, were unable to carry out all previous activities, but were capable of looking after their own affairs without assistance; MRS-3 = moderate disability, that is, they required some help, but could walk without assistance; MRS-4 = moderately severe disability, that is, they were unable to walk without assistance and unable to attend to their own bodily needs without assistance; MRS-5 = had severe disability, that is, they were bedridden, incontinent, and required constant nursing care and attention; and MRS-6 = death at discharge. For prognosis assessment, the patients were divided into 3 groups based on MRS scores: group I, MRS scores of 0–3; group II, MRS scores of 4–5; and group III, an MRS score of 6.

### Laboratory measurements

The MPV value was obtained just after the ED admission, and all blood samples were collected in tubes with ethylenediaminetetraacetic acid (EDTA), which served as the anticoagulant agent and studied within one hour following venipuncture. During the time between venipuncture and processing, the samples were maintained at room temperature. They were studied using optical laser light scatter analysis methods (Abbott, Cell-Dyn Ruby 3700, Chicago, Illinois, USA). Laboratory reference values for MPV were 6.8–10.4 fL.

### Statistical analysis

Data analysis was performed using the Statistical Package for Social Sciences for Windows software, version 11.5 (SPSS Inc., Chicago, IL, United States). Data were shown as mean ± SD or median (min-max) where applicable. The Kolmogorov-Smirnov test was used to determine whether the distributions of continuous variables were normal, while the Student’s t-test, or Mann-Whitney U test was used to analyze mean differences between groups and for comparisons of the medians. Categorical data were analyzed using Pearson’s chi-square or the likelihood ratio test, where applicable, while the mean differences among groups were analyzed using one-way analysis of variance, and the Kruskal-Wallis test was used for comparisons of the medians. Spearman’s rank test was used for correlation analysis. The optimal cut-off points of each clinical variable (for example, latency, hospitalization, MPV and so for), discriminating dead and surviving individuals were evaluated by receiver operating characteristic (ROC) analyses, calculating area under the curve (AUC) as giving the maximum sum of sensitivity and specificity for the relevant test. Sensitivity, specificity, and positive and negative predictive values were also calculated at the best cut-off point for each clinical variable. Determining the best predictors that affect mortality was evaluated by multiple logistic regression analysis. Any variable whose uni variable test had a *p*-value less than 0.25 was accepted as a candidate for the multivariable model along with all variables of known clinical importance. Odds ratios and 95% confidence intervals for each independent variable were also calculated. A *p*-value of <0.05 was considered statistically significant.

## Results

A total 798 consecutive patients who were admitted to the ED with AIS during the study period were retrospectively documented, and finally, 250 eligible patients were included in the study; 126 (50.4%) were male, and 124 (49.6%) were female. The mean age of the patients was 72.3±11.8 (35–97) years. Of the cases, 22 (8.8%) were attended to have been caused by large artery disease, 29 (11.6%) by cardio embolism, 40 (16.0%) by small artery disease, and 159 (63.6%) were of undetermined origin. None of the cases had stroke of other determined etiology. According to the MRS, on discharge from hospital 46 (18.4%) patients had no significant disability (MRS 1); 86 (34.4%) had slight disability (MRS-2); 49 (19.6%) patients had moderate disability (MRS-3); 20 (8.0%) patients had moderately severe disability (MRS-4); and 13 patients (5.2%) had severe disability (MRS-5), 36 (14.4%) patients had died (MRS-6). The median MPV level of all the patients was 8.7 fL (min- max: 4.4–15.7). The demographic and clinical characteristics of all patients are summarized in **Table 1**.

**Table 1:** Demographic and clinical characteristics of all patients (N=250).

Variables	n (%)	
Age (years)	72.3±11.8 (35-97)	
*Gender*		
Female	124	(49.6)
Male	126	(50.4)
*Co-morbidity*		
Hypertension	185	(74.0)
Coronary artery disease	100	(40.0)
Diabetes mellitus	74	(29.6)
Cardiac heart failure	42	(16.8)
Hyperlipidemia	39	(15.6)
Chronic obstructive pulmonary disease	25	(10.0)
Other	138	(55.2)
Smoking	73	(29.2)
Latency	8	(2-120)
*TOAST*		
Large artery disease	22	(8.8)
Cardioembolism	29	(11.6)
Small artery disease (lacune)	40	(16.0)
Stroke of other determined	0	-
Stroke of undetermined	159	(63.6)
*mRS*		
No significant disability	46	(18.4)
Slight disability	86	(34.4)
Moderate disability	49	(19.6)
Moderate-severe disability	20	(8.0)
Severe disability	13	(5.2)
Dead	36	(14.4)
NIHSS	6	(1-18)
Latency (mean)(h)	13.77±17.14 (2-120)	
Hospitalization days	4	(1-116)
TIV(h)	4.0±9.2 (0-65 hours)	
MPV (mean)	8.7 (4.4-15.7)	

SD - standard deviation, min - minimum, max - maximum, TOAST - The Trial Of Org 10172 in Acute Stroke Treatment, mRS - modified Rankin scale, NIHSS - National Institutes of Health Stroke Scale, TIV - time interval of venipuncture, MPV - mean platelet volume

**Figure 1:**
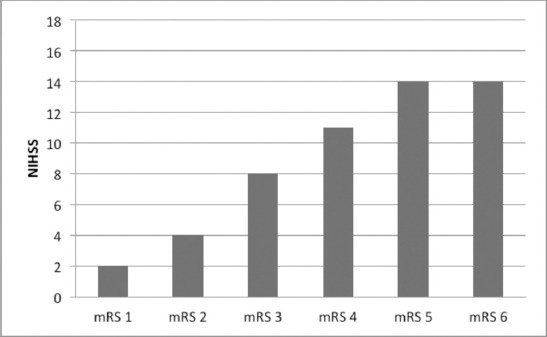
- Shows an increase of mRS scores while NIHSS scores increase. mRS - modified Rankin scale, NIHSS - National Institutes of Health Stroke Scale.

From all of the patients, 36 (14.4%) died and 214 (85.6%) were discharged alive. The male:female ratios of dead and surviving patients were statistically similar (*p*=0.959). The mean age of those who died was statistically significantly higher than those who survived (*p*=0.049), and the median latency of the death group was statistically significantly lower than that of the survivor group (*p*=0.008). There were no statistically significant differences between dead and surviving patients with respect to the median MPV levels (*p*=0.549). The median NIHSS and TOAST scales relating to the patients who died were statistically significantly higher than those relating to the patients who survived (*p*<0.001). There were no statistically significant differences between the dead and surviving patients in terms of median number of hospitalization day *p*=0.842 and TIV *p*=0.549) (**Table 2**). The area under the ROC curves (AUC) regarding latency was statistically significantly different between the survivor and death groups (AUC: 0.638, 95% CI: 0.532–0.744, and *p*=0.008). When distinguishing between the 2 groups, the optimum cut-off value was 6, and the sensitivity, specificity, and positive and negative predictive values of latency were 52.8%, 76.2%, 27.1%, and 90.6%, at this point. The AUC related to hospitalization days, TIV, and MPV measurements were not statistically different between the dead and surviving patients (*p*>0.05). The variables that differed between alive and dead cases were identified as NIHSS, latency, and TOAST. The results of multiple logistic regression analysis are shown in **Table 3**. No statistically significant differences were found between MRS groups (*p*=0.527) and TOAST (*p*=0.797) according to median MPV levels (**Table 4**). There was also no statistically significant correlation between the NIHSS scores and MPV levels of the groups (r=0.017, *p*=0.785). While NIHSS scores increased, MRS scores statistically significantly increased (r=0.899, *p*<0.001) (**Figure 1**). When the clinical and demographical characteristics of patients were considered according to MPV levels divided into 3 groups, there were no statistically significant differences between the 3 groups in terms of age, gender distribution, median latency, TOAST classification, MRS scores distribution, median NIHSS scoring, hospitalization, and TIV. When the clinical and demographical characteristics of patients were considered according to MRS scores divided to 6 subgroups, there were no statistically differences between MRS groups in terms of median latency (*p*=0.087), median hospitalization (*p*=0.394), TIV (h) (*p*=0.201), and MPV levels (*p*=0.847) (**Table 5**).

**Table 2:** Demographic and clinical characteristics for survival status.

Variables	Alive (n=214)		Dead (n=36)		*P*-value
Age (years)	71.7±11.8		75.9±11.4		0.049^†^
*Gender*					0.959^‡^
Female	106	(49.5)	18	(50)	
Male	108	(50.5)	18	(50)	
Latency	8 (2-120)		5 (2-72)		0.008^¶^
NIHSS	4.5 (1-16)		14 (4-18)		<0.001^¶^
*TOAST*					<0.001^$^
Large artery disease	22	(10.3)	-		
Cardioembolism	27	(12.6)	2	(5.6)	
Small artery disease (lacune)	40	(18.7)	-		
Stroke of other determined	-		-		
Undetermined	125	(58.4)	34	(94.4)	0.494^¶^
Hospitalization days	4 (1-36)		4 (1-116)		0.842^¶^
TIV(h)	4.3±9.8		2.1±3.9		0.549
MPV (mean)	8.5 (4.4-13.4)		8.9 (4.8-15.7)		

† Student’s t-test,

‡ Pearson’s Chi-square test,

¶ Mann-Whitney U test,

$ Likelihood ratio test, NIHSS - National Institutes of Health Stroke Scale, TOAST - The Trial Of Org 10172 in Acute Stroke Treatment, TIV - time interval of venipuncture

**Table 3:** The results of multiple logistic regression analysis.

Variables	OR	95% CI	Wald	*P*-value
Age	1.043	0.996-1.093	3.146	0.076
Latency <6	4.867	1.652-14.341	8.239	0.004
NIHSS	1.521	1.327-1.745	36.055	<0.001
TOAST	1.834	1.068-3.150	4.830	0.028

OR - Odds ratio, CI - Confidence interval

**Table 4:** Mean platelet volume levels for TOAST and Groups classifications.

Variables	MPV	*P*-value^[Table-fn t4f1]^
*TOAST*		0.797
Large artery disease	8.9 (5.6-11.9)	
Cardioembolism	8.5 (6.0-13.4)	
Small artery disease (lacune)	8.9 (5.2-12.7)	
Stroke of other determined	-	
Undetermined origin	8.5 (4.4-15.7)	
*Groups*		0.527
Group I (mRS; 1-2-3)	8.6 (4.4-13.4)	
Group II (mRS; 4-5)	8.2 (5.6-12.0)	
Group III (6)	8.9 (4.8-15.7)	

Data shown as median (min-max),

† Kruskal-Wallis test, mRS - modified Rankin scale, MPV - mean platelet volume, TOAST - The Trial Of Org 10172 in Acute Stroke Treatment

**Table 5:** Demographical and clinical characteristics regarding for mRS classification.

Variables	mRS 1 (n=46)		mRS 2 (n=86)		mRS 3 (n=49)		mRS 4 (n=20)		mRS 5 (n=13)		mRS 6 (n=36)		*P*-value
Age (years)	69.6±12.4		71.8±10.7		71.2±14.0		76.7±10.5		72.5±7.4		75.9±11.4		0.107^†^
Gender													0.040^‡^
Female	22	(47.8)	32	(37.2)	31	(63.3)	13	(65)	8	(61.5)	18	(50)	
Male	24	(52.2)	54	(62.8)	18	(36.7)	7	(35)	5	(38.5)	18	(50)	
Latency	8 (2-72)		8.5 (3-120)		8 (2-72)		7.5 (3-72)		8 (3-48)		5 (2-72)		0.087^¶^
NIHSS	2 (1-4)^c,d,e,f,g^		4 (2-12)^a,b,c,h,i^		8 (4-16)^a,d,j,k,l^		11 (8-14)^b,e,j,^		14 (8-16)^f,h,k^		14 (4-18)^g,i,l^		<0.001^¶^
Hospitalization days	4 (1-12)		4 (1-33)		4 (1-15)		4 (1-16)		2.5 (1-36)		4 (1-116)		0.394>^¶^
TIV(h)	4.1±8.6		5.8±12.5		3.6±7.9		1.8±3.7		1.5±4.1		2.1±3.9		0.201^†^
MPV (mean)	8.7 (5.2-12.7)		8.9 (4.4-13.4)		8.4 (5.4-12.3)		8.2 (5.6-11.4)		7.9 (6.4-12.0)		8.9 (4.8-15.7)		0.847^¶^

† One-way ANOVA,

‡ Pearson’s Chi-square test,

¶ Kruskal-Wallis test,

a - mRS 2 vs mRS 3 (*p*<0.05),

b - mRS 2 vs mRS 4 (*p*<0.05),

c - mRS 1 vs mRS 2 (*p*<0.05),

d - mRS 1 vs mRS 3 (*p*<0.001),

e - mRS 1 vs mRS 4 (*p*<0.001),

f - mRS 1 vs mRS 5 (*p*<0.01),

g - mRS 1 vs mRS 6 (*p*<0.01),

h - mRS 2 vs mRS 5 (*p*<0.001),

i - mRS 2 vs mRS 6 (*p*<0.001),

j - mRS 3 vs mRS 4 (*p*=0.023),

k - mRS 3 vs mRS 5 (*p*<0.01),

l - mRS 3 vs mRS 6 (*p*<0.01), mRS - modified Rankin scale, NIHSS - National Institutes of Health Stroke Scale, TIV - time interval of venipuncture, MPV - mean platelet volume, vs – versus

## Discussion

In our short-term FEAIS evaluation, we found that MPV was not associated with overall patient morbidity and mortality. We also found no correlation between different stroke subtypes, NIHSS scores, and MRS scores with regard to MPV levels.

It is thought that MPV is an indicator of platelet activation that is accepted to be associated with systemic inflammatory responses. The relationship between ischemic stroke and MPV has been thoroughly examined in numerous publications. In earlier studies, it was generally accepted that increased platelet activation is related to cerebral infarction and coronary heart disease, while recent reports have shown controversial results regarding the association between MPV and stroke.^6^,^11^ O’Malley et al^12^ reported a higher elevated MPV in patients with acute and chronic ischemic stroke than in controls, but they did not find any significant distinction in the MPV values between the acute and chronic phases of stroke. They also speculated that changes precede the vascular event. Finally, they concluded that MPV is the most important significant variable of the factors associated with stroke; an elevation in MPV level is independently regarded with stroke. Domac et al^8^ reported that a severe stroke had significantly increased MPV levels at admission, reflecting higher platelet reactivity. Muscari et al^13^ observed higher MPV values in arterial stroke with the greatest neurological impairment, while D’Erasmo et al^14^ and Butterworth et al^15^ reported higher platelet volume in patients with AIS than in control individuals. Greisenegger et al^16^ concluded that patients who had suffered an intense stroke attack already had an elevated MPV level, reflecting increased platelet reactivity just before the stroke occurred. Dogan et al^17^ reported enhanced MPV levels in patients with AIS and TIA. They claimed that MPV may provide diagnostic and prognostic information in such a condition. In a study conducted in an emergency room, Furiozzi^18^ found that abnormalities in MPV were a reflection of a preexisting abnormality involved in the primary pathogenesis of the ischemic event and not a secondary phenomenon to cerebrovascular disease. Bath et al^19^ concluded that MPV is increased with AIS, but that the physiological mechanisms that regulate MPV within the megakaryocyte require explanation. Some studies have argued that high MPV is associated with acute cerebral strokes in patients with atrial fibrillation and sinus rhythm.^20^ Li et al^21^ demonstrated that individuals with a high MPV have a higher prevalence of silent cerebral infarction. Ha et al^22^ stated that the patients with MPV higher than 9.4 fL had an up to four-fold elevated risk for cerebral stroke attack. Our findings did not confirm the results of these studies that have speculated that MPV increases occur at acute stroke. In addition, there were no significant differences in terms of MRS scores distribution, median NIHSS scoring, and hospitalization related to MPV. Similarly, we did not find differences among the MRS groups in terms of median latency, median hospitalization, and TIV. Some recent studies have reported results regarding MPV levels and stroke occurrence that are in accordance with our findings. Oz et al^23^ suggested that MPV is not a reliable marker to predict the occurrence of stroke during patient follow up, while Tohgi et al^24^ argued that MPV is significantly lower in patients during the acute and subacute periods of stroke than in controls. Ntaios et al^25^ suggested that platelet volume during the short period (a few days) before stroke does not influence stroke severity. However, some authors; for example, Domac et al,^8^ have claimed a relationship between MPV and a worse outcome. Butterworth et al^15^ claimed that large thrombocyte volume is a risk factor for a poor outcome after ischemic stroke, while Arevalo-Lorido et al^26^ found a relationship between elevated platelet volume and increased dependence rates, as measured by the NIHSS score at admission, which was similar to the findings of Muscari et al.^13^ They eventually concluded that there is an increase in morbidity and cardiovascular mortality in patients with high MPV after suffering a stroke. Greisenegger et al^16^ concluded that increased MPV was associated with a worse outcome in patients suffering an AIS event, while Pikija et al^9^ speculated that higher MPV is associated with a greater risk of death/dependence 7 days post-stroke. Shah et al^27^ reported that patients with higher MPV had a worse outcome after one week; independent of stroke subtype, and Zhang et al^6^ found that higher MPV is associated with higher mortality rate. Our findings showed no correlation between MPV and MRS scores, but MRS scores generally increased while NIHSS scores increase. Furthermore, we could not show any correlation between worst outcome and elevated MPV levels. In accordance with our data, Ntaios et al^28^ concluded that MPV is not related to stroke severity or functional outcome, and does not differ between stroke subtypes. Similarly, O’Malley et al^12^ found no association between platelet volume and prognosis.

Several plausible explanations may account for these contradictions between studies; first, these studies probably failed to reveal time-dependent artificial elevations in MPV due to the platelet-swelling that is observed following both EDTA and citrate anticoagulant incubation, and which is more prominent with regard to EDTA anticoagulants.^5^,^12^,^29^,^30^ Some recent studies have found that this elevation of platelet volume amounts to less than 0.5 fL when the waiting interval is less than 2 hours after venipuncture.^8^,^16^,^29^-^31^ Second, the samples were measured with different automated cell counters, which may vary in their methods, hence, the accuracy of MPV measurements may be influenced by the platelet-counting method of the analyzers and can show poor agreement. Conventional cell analyzers, which use light scattering or impedance, may conduce to this relatively poor result, especially in the thrombocytemic and/or thrombocytopenic conditions.^7^,^25^,^32^,^33^ Third, MPV measurement was performed at different time intervals in the different studies, and venipuncture ranged from admission to 48 hours or longer following stroke onset.^13^,^15^,^16^ The average lifetime of the platelet is approximately 8 days; the elevated MPV is measured within the first 48 hours following AIS. Time between stroke initiation and blood sampling can affect the determined parameter.^9^,^12^,^16^,^25^,^32^ Fourth, different factors, such as ethnicity, age, genetic factors, communal settings, and instruments that are used to assess normal MPV values, were not considered; hence, these conditions may have affected the MPV estimations, and erroneous interpretations of results could have been made. Some studies have suggested that each laboratory should establish its own reference ranges.^34^-^36^ Another potential reasonable explanation for these divergent results could be study size; the majority were of small or modest sizes and covered varying clinic periods. Finally, it should be noted that not all studies used the same score to assess outcome. In addition, standardized methods must be used in MPV measurement. It might then be worthwhile defining a local normal range with healthy volunteers across the entire age range.^16^,^25^,^29^,^35^

### Limitations

The present study has several limitations. First, it was retrospective in nature. The data volume of an electronic medical record system is always very large; therefore, missing data is prevalent and this may introduce bias. One solution is to handle missing data with simple imputation or multiple imputations. Multiple imputations are an advanced technique for handling missing values. It is superior to single imputation in that it takes into account uncertainty in missing value imputation. This study has a limitation associated with the analysis methods of missing data. Therefore, there is a need for future studies including analysis methods of missing data, in order to decrease possible bias in the study.^36^-^38^ Additionally, we could not obtain regular control blood samples several days later to compare the prognostic significance of early and late MPV measurements, although these were not actually included among the objectives of this investigation. Second, we did not analyze infarct size and location. Thus, there may be an association between a specific lesion location and its size and MPV scores. Third, the study lacks information regarding inflammatory and other markers, such as those of thrombotic and fibrinolytic status, activation, and aggregation factors, which may have affected the results. Finally, we only compared MPV values between stroke subgroups accepted as controls, rather than using a healthy control group; therefore, we cannot know whether these MPV values are the same as those that would be obtained from healthy individuals.

In conclusion, our results suggested that MPV is not a reliable marker for mortality and prediction of prognosis or functional outcome of FEAIS attack in a short-term follow-up period. Other factors that may play a role in stroke prognosis, such as a proinflammatory state, an inflammatory process, hormones, a prothrombotic condition, and aggregation markers, or a platelet membrane protein that increases platelet activation, and vascular premorbidity, remain unclear.
